# The Value of Oxygenation Saturation Index in Predicting the Outcomes of Patients with Acute Respiratory Distress Syndrome

**DOI:** 10.3390/jcm7080205

**Published:** 2018-08-08

**Authors:** Wan-Ling Chen, Wei-Ting Lin, Shu-Chen Kung, Chih-Cheng Lai, Chien-Ming Chao

**Affiliations:** 1 Department of Respiratory Therapy, Chi Mei Medical Center, Liouying, Tainan 73657, Taiwan; wanlin0810@gmail.com (W.-L.C.); clh7810@mail.chimei.org.tw (S.-C.K.); 2 Departments of Orthopedics and Trauma, Chi Mei Medical Center, Tainan 71004, Taiwan; aapriliaa@gmail.com; 3 Department of Intensive Care Medicine, Chi Mei Medical Center, Liouying, Tainan 73657, Taiwan; dtmed141@gmail.com

**Keywords:** SpO_2_/FiO_2_, acute respiratory distress syndrome, mortality, PaO_2_/FiO_2_

## Abstract

This study aims to investigate the association between oxygenation saturation index (OSI) and the outcome of acute respiratory distress syndrome (ARDS) patients, and assess the predictive performance of OSI for ARDS patients’ mortality. This study was conducted at one regional hospital with 66 adult intensive care unit (ICU) beds. All patients with ARDS were identified between November 1 2016 and May 31 2018, and their clinical information was retrospectively collected. The lowest PaO_2_/FiO_2_ ratio and SpO_2_/FiO_2_ ratio and highest mean airway pressure (MAP) were recorded on the first day of ARDS; and oxygen index (OI) and OSI were calculated as (FiO_2_ × MAP × 100)/PaO_2_, and (FiO_2_ × MAP × 100) /SpO_2_ accordingly. During the study period, a total of 101 patients with ARDS were enrolled, and their mean age was 69.2 years. The overall in-ICU and in-hospital mortality rate was 57.4% and 61.4%, respectively. The patients with in-ICU mortality had higher APACHE II score than the survivors (31.6 ± 9.8 vs. 23.0 ± 9.1, *p* < 0.001). In addition, mortalities had lower SpO_2_, and SpO_2_/FiO_2_ ratios than the survivors (both *p* < 0.05). In contrast, survivors had lower OI, and OSI than the mortalities (both *p* = 0.008). Both OSI (area under curve (AUC) = 0.656, *p* = 0.008) and OI (AUC = 0.654, *p* = 0.008) had good predictive performance of mortality among ARDS patients using receiver-operating characteristics (ROC) curves analysis. In addition, the AUC of SpO_2_/FiO_2_ (AUC = 0.616, *p* = 0.046) had better performance for mortality prediction than PaO_2_/FiO_2_ (AUC = 0.603, *p* = 0.08). The patients with OSI greater than 12 had a higher risk of mortality than OSI < 12 (adjusted OR, 5.22, 95% CI, 1.31–20.76, *p* = 0.019). In contrast, OI, PaO_2_/FiO_2_, and SpO_2_/FiO_2_ were not found to be significantly associated with increased mortality. OSI is significantly associated with the increased mortality of ARDS patients and can also be a good outcome predictor.

## 1. Introduction

Acute respiratory distress syndrome (ARDS) is an acute catastrophic lung condition that can be associated with high mortality [1,2,3]. In 1994, the American and European Consensus Conference (AECC) established specific clinical criteria for ARDS and acute lung injury (ALI), including acute and sudden onset of severe respiratory distress, bilateral infiltrates on chest radiography, the absence of cardiogenic pulmonary edema, and severe hypoxemia [4]. Until 2012, the Berlin definition of ARDS was proposed as three categories of ARDS based on the degree of hypoxemia: mild (200 mm Hg < PaO_2_/FiO_2_ ≤ 300 mm Hg), moderate (100 mm Hg < PaO_2_/FiO_2_ ≤ 200 mm Hg) and severe (PaO_2_/FiO_2_ ≤ 100 mm Hg) with a PEEP ≥ 5 cm H_2_O [5]. No matter which criterion is applied for ARDS, severe hypoxemia remains the hallmark of ARDS. Therefore, various parameters of oxygenation are assessed as appropriate indicators of disease severity or good predictors of outcomes in several studies [6,7,8,9]. However, accurate analysis of oxygenation relies on the arterial blood gas measurement, and this test requires repeated arterial blood gas sampling. Thus, the concerns about the complications of arterial blood gas tests, including excess blood draws, increasing medical cost, and the implementation of arterial lines are raised. If we want to obtain accurate oxygenation measurement and avoid the arterial blood gas-associated complications, pulse oximetric measurement of oxygenation saturation (SpO_2_) may provide a solution.

Through the help of noninvasive pulse oximetry, clinicians can obtain SpO_2_ and calculate the SpO_2_/FiO_2_ ratio, which has been demonstrated to be well correlated with the PaO_2_/FiO_2_ ratio among patients with mechanical ventilation and ARDS after using a nonlinear imputation strategy [10,11]. Moreover, DesPrez et al. showed that oxygenation saturation index (OSI) combining the SpO_2_/FiO_2_ ratio and mean airway pressure was well correlated with the oxygenation index (OI) and could be a reliable predictor of ARDS patients’ outcome [12]. However, the accuracy of SpO_2_ measurement may be affected by several factors, such as hypoperfusion, abnormal hemoglobin, severe anemia, or use of vasopressor, and we wonder whether OSI based on the measurement of SpO_2_ can perform well in all of the various clinical conditions in ARDS. Therefore, we conduct this retrospective study to investigate the association between OSI and the outcome of ARDS patients, and assess the predictive performance of OSI for ARDS patients’ mortality.

## 2. Patients and Methods

### 2.1. Patients and Hospital Setting

This study was conducted at one regional hospital with 66 adult adult intensive care unit (ICU) beds. The care in the ICU is conducted by ICU teams, including intensivists, senior residents, nurses, respiratory therapists, dietitians, physical therapists, and clinical pharmacists. The ICU team makes rounds at least once daily, and respiratory therapists are responsible for managing all mechanical ventilation (MV), including weaning processes and spontaneous breathing trials. Patients who were admitted to ICUs and met the American-European Consensus Conference (AECC) definition of ARDS were identified between 1 November 2016 and 31 May 2018. Ethics approval was obtained from the Institution Review Board of Chi Mei Medical Center.

### 2.2. Variables Measurements

The following information of the included patients, including age; gender; co-morbidities; cause of ARDS; clinical features; laboratory data; comorbidities including congestive heart failure, chronic lung diseases, chronic kidney disease, liver cirrhosis, diabetes mellitus, cancer, and immunocompromised condition; and Acute Physiology and Chronic Health Evaluation (APACHEII) score were collected. The primary outcome was all-cause in-ICU mortality, and secondary outcomes included in-hospital mortality, length of ICU and hospital stays, and MV duration. In addition, the results of arterial blood gas (ABG); SpO_2_ and ventilator setting including FiO_2_, tidal volume, positive end-expiratory pressure (PEEP), and mean airway pressure (MAP) were obtained. In addition, the lowest PaO_2_/FiO_2_ ratio and SpO_2_/FiO_2_ ratio were recorded on the first day of ARDS. SpO_2_ was used to calculate oxygenation saturation index and SpO_2_/FiO_2_ ratio if the SpO_2_ < 97% as previous study [12].

### 2.3. Definitions

The shock was defined as systolic blood pressure (SBP) of ≤90 mmHg or a mean arterial pressure (MAP) ≤65 mmHg for at least 1 hour despite adequate fluid resuscitation; or the need for vasoactive agents to maintain SBP ≥90 mmHg or mean arterial pressure ≥65 mmHg. ARDS was diagnosed according to AECC criteria—severe hypoxemia including PaO_2_/FiO_2_ ratio less than 200 mmHg, bilateral infiltrates on chest X-ray, and no evidence of cardiogenic pulmonary edema [4]. Oxygen index (OI) was defined as (FiO_2_ × MAP x 100) divided by PaO_2_, and Oxygenation saturation index (OSI) was defined as (FiO_2_ × MAP × 100) divided by SpO_2_.

### 2.4. Statistical Analysis

Continuous variables were reported as the mean and standard deviation (SD). Categorical variables were presented as frequency counts with percentages. In addition, the differences of baseline characteristics and clinical variables between the survival and non-survival groups were evaluated using Student’s test (for continuous variables) and Pearson’s chi-squared test (for categorical variables). Receiver-operating characteristics (ROC) curves were used to assess the predictive value of different severity indexes for the outcome of ARDS patients and to determine the best cut off values of OSI and OI for the outcome prediction. All statistical analyses were conducted using the statistical package SPSS for Windows (Version 19.0, SPSS, Chicago, IL, USA), and a *p* value < 0.05 was considered to show statistical significance.

## 3. Results

During the study period, a total of 101 patients with ARDS were enrolled, and their mean age was 69.2 years. The mean APACHE II scores were 27.9 ± 10.4. Only eight patients had extra-pulmonary ARDS. Pneumonia was the most common cause of ARDS (*n* = 94, 94.1%), followed by sepsis (*n* = 5, 5.0%). In addition, each patient had major trauma and acute pancreatitis-related ARDS. Among them, 81 (80.2%) patients had an initial presentation of shock. Malignancy was the most common underlying diseases (*n* = 42, 41.6%), followed by diabetes mellitus (*n* = 33, 32.7%), chronic kidney disease (*n* = 21, 20.8%), liver cirrhosis (*n* = 11, 10.9%), congestive heart failure (*n* = 8, 7.9%), and chronic lung disease (*n* = 5, 5.0%). The median hemoglobin and total-bilirubin was 9.9 g/dL (8.8–11.4) and 1.0 mg/dL (0.5–2.0), respectively. The median MV duration was 12.0 days (5–21.8). The overall in-ICU and in-hospital mortality rate was 57.4% and 61.4%, respectively. The median length of stay in ICU and hospital was 13.0 days (4.3–25.8) and 18.0 days (4.3–33.3), respectively. 

The patients had in-ICU mortality had higher APACHE II score than the survivors (31.6 ± 9.8 vs. 23.0 ± 9.1, *p* < 0.001). In addition, the patients with mortality had lower SpO_2_ and SpO_2_/FiO_2_ ratios than the survivors (both *p* < 0.05). In contrast, survivors had lower OI, and OSI than the mortalities (both *p* = 0.008). However, there were no significant differences in terms of age, gender, the cause of ARDS, the presence of shock, underlying disease and the findings of laboratory examinations between non-survivors and survivors (Table 1).

**Figure 1:**
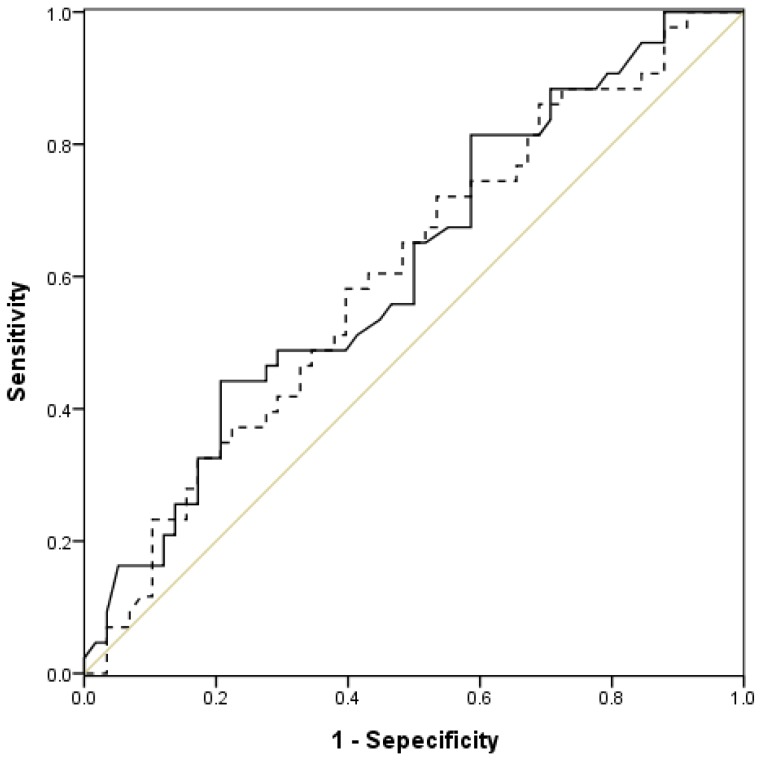
Receiver-operative characteristics (ROC) curves of oxygenation saturation index (continuous line) and oxygenation index (dotted line) for mortality prediction.

**Figure 2:**
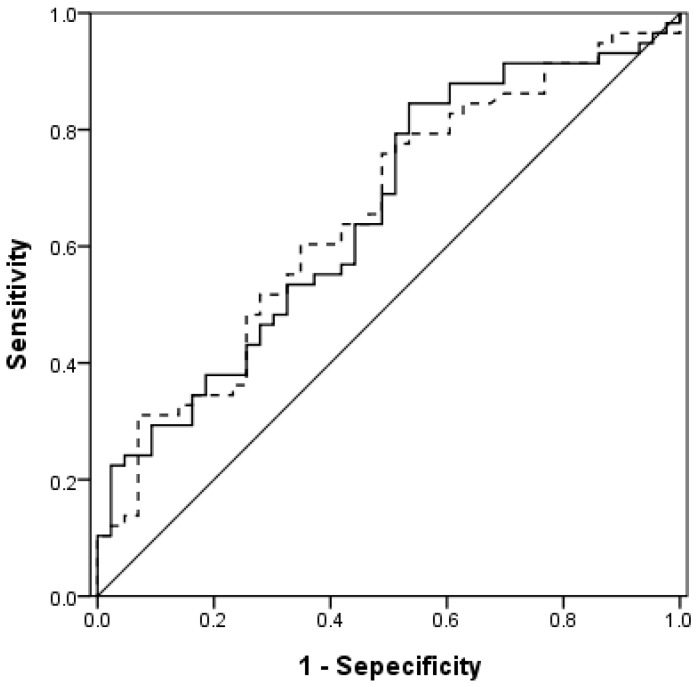
Receiver-operative characteristics (ROC) curves of SpO_2_/FiO_2_ (continuous line) and PaO_2_/FiO_2_ (dotted line) for mortality prediction.

Using the ROC curve, we assessed the individual performance of OI, OSI, PaO_2_/FiO_2_, and SpO_2_/FiO_2_ to predict in-ICU mortality (Table 2). Both OSI (AUC = 0.656, *p* = 0.008) and OI (AUC = 0.654, *p* = 0.008) had good predictive performance of mortality among ARDS patients (Figure 1), and the best cutoff value for OSI and OI was 12 and 16, respectively. In addition, the AUC of SpO_2_/FiO_2_ (AUC = 0.616, *p* = 0.046) had better performance for mortality prediction than PaO_2_/FiO_2_ (AUC = 0.603, *p* = 0.08) (Figure 2). The best cutoff value for SpO_2_/FiO_2_ ratio was 142. Furthermore, we analyzed the association between mortality of ARDS patients and these four indices—OI, OSI, PaO_2_/FiO_2_, and SpO_2_/FiO_2_ (Table 3). We found only OSI were significantly associated with increased mortality. The patients with OSI greater than 12 had the higher risk of mortality than OSI < 12 (adjusted OR, 5.22, 95% CI, 1.31–20.76, *p* = 0.019). In contrast, OI, PaO_2_/FiO_2_, and SpO_2_/FiO_2_ were not found to be significantly associated with increased mortality. 

## 4. Discussion

In this study, we found that OSI was significantly associated with increased mortality of ARDS patients. Moreover, it had a similar performance for mortality prediction to OI, but better than the other two oxygenation parameters—PaO_2_/FiO_2_, and SpO_2_/FiO_2._ Based on this finding, it suggests that OSI as a noninvasive parameter can provide useful information of ARDS outcome. This is consistent with DesPrez et al.’s findings [12] that OSI was independently associated with hospital mortality (OR, 1.228; 95% CI, 1.056–1.429), and the AUC for mortality prediction was greater for OSI (AUC, 0.602; *p* = 0.007) than the other three oxygenation parameter—OI, PaO_2_/FiO_2_, and SpO_2_/FiO_2._ In contrast, the clinical characteristics of ARDS patients in the present study were significantly different from DesPrez et al.’s study. Most of the patients were elderly, aged ≥65 years in our study. In addition, 82 (81.2%) patients had a hemoglobin <12 g/dL, and 81 (80.2%) patients had shock in this study. Although the accuracy of pulse oximeter may be influenced by some clinical conditions, such as hypoperfusion and anemia, our findings indicated that OSI could preserve fair performance in the outcome prediction of ARDS patients in the patients with shock or elderly patients. Finally, we found that the OSI cutoff value of 12 had the best predictive performance and the patients with OSI ≥ 12 had five times the risk of death than the patients with OSI < 12. Overall, it should suggest that OSI could be a good prognostic indicator of ARDS patients.

Besides the comparison between OSI and OI, we recorded PaO_2_/FiO_2_, and SpO_2_/FiO_2_ for comparison. According to ROC curve analysis, we found that the predictive performance of PaO_2_/FiO_2_, and SpO_2_/FiO_2_ were similar. This is consistent with Brown et al.’s study [10] that mortalities of ARDS patients were similar on the bases of measured PaO_2_/FiO_2_ and SpO_2_/FiO_2_. Even for diagnosing ARDS, Chen et al. [13] showed that the clinical characteristics and outcome of ARDS patients diagnosed by SpO_2_/FiO_2_ were similar compared with diagnosed by PaO_2_/FiO_2_. In summary, SpO_2_, which is non-invasively measured by the pulse oximeter and can provide the continuous monitor of oxygenation, can be considered as a good surrogate of ARDS mortality compared to PaO_2_ which requires sampling of arterial blood for analysis. 

No matter in the Berlin definition [5] or AECC criteria [4], PaO_2_/FiO_2_ was an important indicator for diagnosing ARDS and was commonly used to represent the severity of ARDS. Although the Berlin criteria showed the better predictive power of mortality in ARDS than the AECC definition, the absolute value of AUC on the ROC analysis was only 0.577 [14]. Another prospective observation study also found that ARDS severity was not significantly associated with the mortality in the Cox proportional hazard regression analysis (*p* = 0.84) [15]. In fact, more and more studies [9] showed that PaO_2_/FiO_2_ may not be a good prognostic factor of ARDS. In this study, PaO_2_/FiO_2_ was not found to be significantly associated with the mortality of ARDS patients and showed the poor predictive performance of OI using ROC analyses. 

This study had several limitations. First, this study was conducted in a single medical center, and the patient number was limited. Therefore, our findings may not be generalized to other hospitals. Second, ARDS can be caused by many diseases; however, more than 90% of ARDS cases in this study were caused by pneumonia. Third, we do not have the cases of carbon monoxide poisoning, methemoglobin intoxication or hyperbilirubinemia, which may affect the measurement of the pulse oximeter. Overall, a further large and multicenter study is warranted to confirm our findings.

In conclusion, OSI is significantly associated with the increased mortality of ARDS patients and can also be a good outcome predictor.

## Figures and Tables

**Table 1:** Clinical characteristics of patients with ARDS.

Variables	No (%) of Patients (*n* = 101)	No (%) of Survivors (*n* = 43)	No (%) of Mortalities (*n* = 58)	*p* Value *
Age (years)	69.2 ± 13.5	66.7 ± 14.4	71.0 ± 12.7	0.12
Gender				
Male	63 (62.4)	25 (58.1)	38 (65.5)	0.45
Female	38 (37.6)	18 (41.9)	20 (34.5)	
APACHE II scores	27.9 ± 10.4	23.0 ± 9.1	31.6 ± 9.8	<0.001
Extra-pulmonary ARDS	8 (7.9)	3 (7.0)	5 (8.6)	0.76
Cause of ARDS				
Pneumonia	94 (94.1)	40 (93.0)	54 (93.1)	0.29
Sepsis	5 (5.0)	1 (2.3)	4 (6.9)	
Major trauma	1 (1.0)	1 (2.3)	0 (0)	
Acute pancreatitis	1 (1.0)	1 (2.3)	0 (0)	
Shock	81 (80.2)	32 (74.4)	49 (84.5)	0.21
Underlying disease				
Chronic lung disease	5 (5.0)	1 (2.3)	4 (6.9)	0.30
Chronic kidney disease	21 (20.8)	10 (23.3)	11 (19.0)	0.60
Congestive heart failure	8 (7.9)	2 (4.7)	6 (10.3)	0.30
Liver cirrhosis	11 (10.9)	5 (11.6)	6 (10.3)	0.84
Diabetes mellitus	33 (32.7)	15 (34.9)	18 (31.0)	0.68
Malignancy	42 (41.6)	15 (34.9)	27 (46.6)	0.24
Ventilator setting				
PEEP	10 (10–12)	10 (10–12)	10 (10–12)	0.47
Vt	450 (410–549)	476 (420–557)	450 (400–534)	0.13
Laboratory findings				
Hemoglobin	9.9 (8.8–11.4)	9.9 (9.7–17.2)	9.9 (9.2–10.9)	0.96
Total-bilirubin	1.0 (0.5–2.0)	0.8 (0.5–1.6)	1.1 (0.6–2.4)	0.16
pH	7.35 (7.28–7.41)	7.35 (7.28–7.41)	7.34 (7.28–7.42)	0.98
HCO_3_^−^	20.3 (16.9–24.1)	20.3 (17.0–22.5)	20.0 (16.4–26.1)	0.91
Lowest PaO_2_	72.0 (64.2–83.1)	72.2 (64.3–83.2)	71.7 (63.4–83.4)	0.71
Lowest SpO_2_	90.0 (85.5–92.5)	92.0 (90.0–94.0)	88.0 (80.8–91.3)	<0.001
Lowest PaO_2_/FiO_2_ ratio	108.8 (91.7–138.3)	114.0 (100.9–145.6)	106.0 (88.7–130.0)	0.08
Lowest SpO_2_/FiO_2_ ratio	145.0 (116.3–186.0)	148.3 (130.7–190.0)	139.3 (102.1–163.5)	0.046
Highest mean airway pressure	21.0 (19.5–23.0)	21.0 (18.0–22.0)	21.5 (20.0–24.0)	0.012
Oxygenation index	19.0 (15.0–23.5)	15.9 (13.7–22.1)	20.5 (16.0–26.6)	0.008
Oxygenation saturation index	15.0 (11.3–18.2)	13.6 (9.7–17.2)	16.0 (12.9–21.4)	0.008
Outcome				
MV duration	12.0 (5–21.8)	15.5 (8.8–32.5)	8.0 (3.4–19.0)	0.002
ICU LOS	13.0 (4.3–25.8)	19.0 (10.8–38)	7.0 (2.8–18.0)	<0.001
Hospital LOS	18.0 (4.3–33.3)	32.5 (19.5–48.5)	7.0 (2.8–18.0)	<0.001

* Comparison between survivors and mortalities.

**Table 2:** Comparison of areas under the receiver-operating characteristic (ROC) curve for discrimination of mortality of ARDS for four indices of oxygenation.

Measure	Area under the ROC Curve	95% CI	*p* Value
Lowest PaO_2_/FiO_2_ ratio	0.603	0.492–0.714	0.08
Lowest SpO_2_/FiO_2_ ratio	0.616	0.506–0.726	0.046
Oxygenation index	0.654	0.547–0.761	0.008
Oxygen saturation index	0.656	0.548–0.763	0.008

**Table 3:** Risk factor of in-ICU mortality.

Variable	OR	95% CI	*p* Value
Lowest PaO_2_/FiO_2_ ratio			
≤100	1		
>100	0.794	0.25–2.52	0.7
Lowest SpO_2_/FiO_2_ ratio			
≤142	1		
>142	0.36	0.09–1.35	0.13
Oxygenation index			
<16	1		
≥16	2.92	0.98–8.68	0.054
Oxygenation saturation index			
<12	1		
≥12	5.22	1.31–20.76	0.019

OR, odds ratio; CI = confidence interval.
